# Hepatotoxicity Modeling Using Counter-Propagation Artificial Neural Networks: Handling an Imbalanced Classification Problem

**DOI:** 10.3390/molecules25030481

**Published:** 2020-01-23

**Authors:** Benjamin Bajželj, Viktor Drgan

**Affiliations:** 1 National Institute of Chemistry, Hajdrihova 19, 1001 Ljubljana, Slovenia; benjamin.bajzelj@ki.si; 2 Biotechnical Faculty, University of Ljubljana, Jamnikarjeva 101, 1000 Ljubljana, Slovenia

**Keywords:** hepatotoxicity, counter-propagation artificial neural networks, imbalanced dataset, genetic algorithm, QSAR

## Abstract

Drug-induced liver injury is a major concern in the drug development process. Expensive and time-consuming *in vitro* and *in vivo* studies do not reflect the complexity of the phenomenon. Complementary to wet lab methods are *in silico* approaches, which present a cost-efficient method for toxicity prediction. The aim of our study was to explore the capabilities of counter-propagation artificial neural networks (CPANNs) for the classification of an imbalanced dataset related to idiosyncratic drug-induced liver injury and to develop a model for prediction of the hepatotoxic potential of drugs. Genetic algorithm optimization of CPANN models was used to build models for the classification of drugs into hepatotoxic and non-hepatotoxic class using molecular descriptors. For the classification of an imbalanced dataset, we modified the classical CPANN training algorithm by integrating random subsampling into the training procedure of CPANN to improve the classification ability of CPANN. According to the number of models accepted by internal validation and according to the prediction statistics on the external set, we concluded that using an imbalanced set with balanced subsampling in each learning epoch is a better approach compared to using a fixed balanced set in the case of the counter-propagation artificial neural network learning methodology.

## 1. Introduction

The liver is the primary site of xenobiotic metabolism, and as such, is prone to suffering from toxic effects. Moreover, it is well known that drug-induced liver injury (DILI) poses one of the main challenges in the drug development process. Aside from efficacy, toxicity is the main reason for the termination of drug development [1], while hepatotoxicity is one of the biggest threats in the drug development process. Hepatotoxicity presents the main reason for the withdrawal of drugs from the market [2,3]. Therefore, it is important to address the problem in the early stages of drug development. Preclinical stages consist of obligatory animal testing. However, a study on 3290 approved drugs and formulations [4] suggests that the absence of hepatotoxic effects in animals does not generally predict safety in humans. The study also implies medium predictive power of a positive result on animals in the case of liver disorders. Apart from moderate predictivity, other reasons aimed at reducing animal testing are ethical concerns and implementation of the reduction, refinement, and replacement (“3R”) strategy in research [5]. Human primary hepatocytes (hPH) are considered the gold standard for studying *in vitro* hepatotoxicity, but their major limitation is that they rapidly de-differentiate and liver-specific functions, such as albumin production and cytochrome P450 expression, decline quickly over the first 24–48 h of culture [6]. The use of immortalized primary hepatocytes, which suffer from hindered biotransformation capabilities, is frequent. Alternatives to *in vitro* toxicity include induced pluripotent stem cells [7] (especially in high throughput screening), while more complex models are being developed, such as organoids or liver-on-a-chip [8,9]. *In vitro* methods evaluating endpoints related to DILI can achieve a sensitivity of up to 70% [10]. However, while they substitute animal testing, *in vitro* methods are not truly equal to the *in vivo* system and may also be time consuming and expensive. In this regard, *in silico* methods offer a time- and cost-efficient method to address the issue of toxicity early in the drug development process [11]. In recent years, efforts have been made to predict hepatotoxicity using various techniques of quantitative structure–activity relationship (QSAR) modeling. QSAR studies of DILI are based mainly on small datasets. One such study was conducted by Cruz-Monteagudo [12]. They achieved good results using 3D molecular descriptors and a training dataset with 33 compounds in the hepatotoxic class and 41 compounds in the non-hepatotoxic class. In their study, based on a structurally and pharmacologically diverse training dataset, they developed classification models using linear discriminant analysis (LDA), artificial neural networks using radial basis function (RBF) architecture, and the OneR classification algorithm. In the external validation experiment, they achieved sensitivity of 100% and specificity of 67% using the LDA model, sensitivity of 67% and specificity of 67% using the RBF model, and sensitivity of 67% and specificity of 100% using the OneR classifier. A study with a larger number of compounds was conducted by Ekins et al. [13]. In the study, they used extended connectivity fingerprint counts along a few additional interpretable 2D descriptors for the Bayesian model. Sensitivity and specificity on the external set of 237 compounds were 56% and 67%, respectively. On a smaller set of 28 structurally similar compounds with different DILI potential, the performance was similar. Fourches et al. [14] developed QSAR models with support-vector machines (SVMs) for hepatotoxicity, with a dataset of 531 compounds (248 hepatotoxic, 283 non-hepatotoxic) acquired with text mining, and achieved accuracy from 55.7% to 72.6% with external cross-validation. As discussed in Kotsampasakou et al. [15], data curation is of high importance and text mining is error-prone and presents the reason for a lower quality of dataset. More accurate predictions can be made using ensemble modeling, as was shown in the study of Liew et al. [10]. The ensemble model, constructed by the stacking of random forest, k-nearest neighbor, and naive Bayes models with naive Bayes, was based on 2D descriptors. While the average sensitivity and specificity of 617 base classifiers on the external set of 120 compounds were 62.4% and 61.8%, respectively, the ensemble model had 84.5% sensitivity and 65.1% specificity for 101 objects of the external set that were inside the applicability domain. It is worth noting, however, that the ensemble model could not separate non-toxic compounds from structurally very similar toxic compounds, the problem also encountered in the study of Ekins et al. [13]. The study also suggested that weakly hepatotoxic compounds have a big influence on decision boundary and their removal greatly affects prediction metrics. In the recent study by Wang et al. [16], which used 450 molecules, an ensemble model was built mainly on different molecular fingerprints using multiple machine learning algorithms, and achieved an accuracy of 81.67%, sensitivity of 64.55%, specificity of 96.15%, and the area under the receiver operating characteristic curve (AUC) of 80.35% on the external validation set. A recursive random forest approach on a set containing 122 DILI-positive and 932 DILI-negative compounds with adjusting decision threshold was used in the study by Zhu et al. [17] to achieve good sensitivity and specificity (both around 80%) for models each using different types of structure presentation (CDK, MACCS, and Mold2 descriptors). In that way, the authors significantly reduced the number of meaningful descriptors, as they ended up with 8, 26, and 20 descriptors. In this study, ensemble modeling also significantly improved performance.

In our study, we employ counter-propagation artificial neural networks (CPANNs) in an attempt to capture the nonlinear relationship between molecular structure and hepatotoxicity of drugs. CPANNs combine supervised and unsupervised learning strategies and have been successfully applied to different QSAR problems [18,19,20]. Due to the learning strategies used, they are especially suitable for classification problems. To our knowledge, the method has never been used for modeling hepatotoxicity before. The majority of the drugs in the data set used do not exhibit hepatotoxicity. To deal with the imbalanced dataset, we apply two approaches. One approach presents manual balancing of the training dataset prior to model building with CPANNs. In the second approach, we modify the classical training algorithm of CPANNs by integrating random subsampling into the training procedure to obtain balanced representation of two toxicity classes during the training of a CPANN. Molecular descriptors calculated from 2D representation of compounds are used in optimizations of CPANN models by a genetic algorithm. With 148 models passing internal validation criteria, we construct two consensus models, one using 24 models and the other one using 124 models. The first consensus model, based on a manually-constructed balanced training dataset, yields sensitivity and specificity of 0.5 and 0.79, respectively. The second consensus model, obtained using models developed by the modified training algorithm, has sensitivity and specificity of 0.65 and 0.85, respectively. A simple metric is constructed to identify descriptors important for the prediction of hepatotoxicity.

## 2. Results and Discussion

### 2.1. Dataset

**Figure 1:**
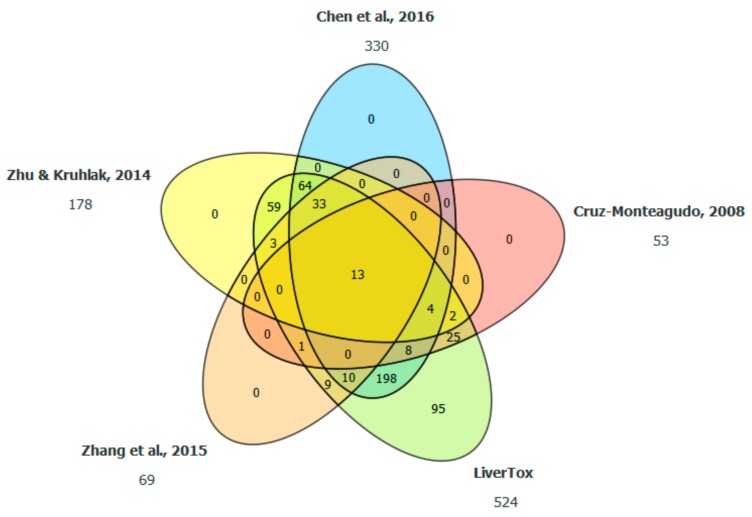
Venn diagram of selected compounds. Compounds in our dataset that we classified either as DILI-positive or DILI-negative were extracted from different literature sources. Only LiverTox contained compounds that were not present in any of the other sources.

After data curation, our dataset contained 524 compounds, which were gathered using five literature sources. As can be seen in Figure 1, there were no compounds that were selected from just one source; an exception is the LiverTox database.

**Figure 2:**
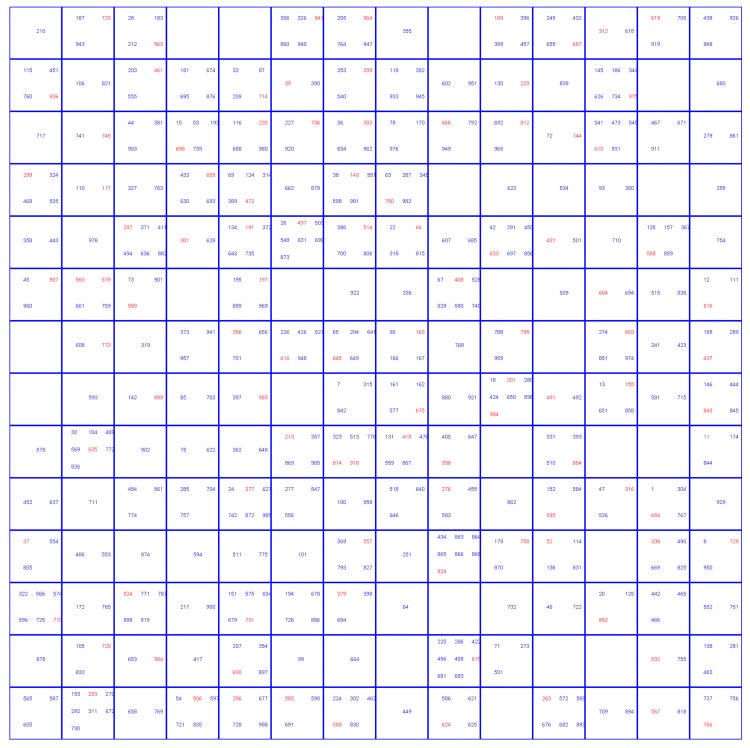
Selection of validation set compounds for the first approach using the Kohonen top-map.

**Figure 3:**
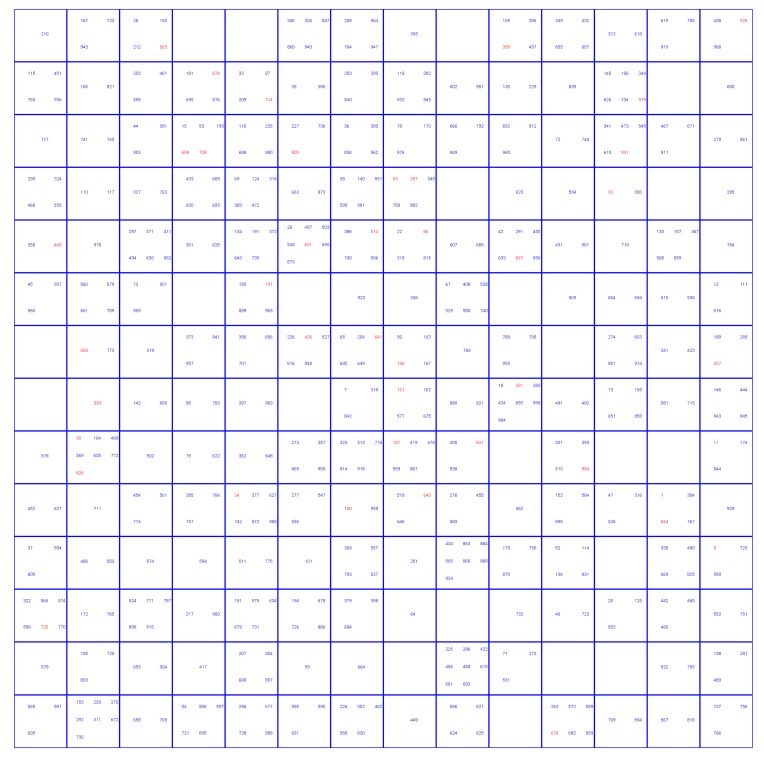
Selection of validation set compounds for the second approach using the Kohonen top-map.

Different approaches that were used to deal with dataset imbalance influenced the selection of compounds into training set, test set (TE1), internal validation set (TE2), and external validation set. The underlying reason was the difference in the size of training sets for the two approaches. In the first approach, a balanced number of hepatotoxic and non-hepatotoxic compounds was used in the training set, which consisted of 216 compounds. For the second approach, where the modified CPANN training algorithm was applied, an imbalanced training set of 404 compounds, with 26.7% compounds in the hepatotoxic class, was used. Thus, the test set, internal validation set, and external validation set used in the first approach contained a larger number of compounds from the non-hepatotoxic class. The class distribution of compounds among the sets is given in Table 1. The selection of compounds into the validation set was made based on the distribution of compounds on the Kohonen top-map shown in Figure 2 and Figure 3 for the first and the second approach, respectively. The red color in Figure 2 and Figure 3 indicates compounds selected for the validation set. Principal component analysis (PCA) was performed to verify if it could separate compounds belonging to the hepatotoxic and non-hepatotoxic classes. It was observed that the first few principal components could not separate the two classes of compounds. More details are given in the Supplementary Materials (Supplementary file: Supplementary1.pdf).

### 2.2. Models

Optimizations of CPANNs were made using the manually balanced training set in the first approach and using the modified CPANN training algorithm in the second approach. The optimizations for the first approach resulted in 24 models that passed our criteria, i.e., with sensitivity and specificity on training, test set TE1, and test set TE2 of at least 0.7, where test set TE2 was used as the internal validation set. The second approach resulted in 124 models passing the same criteria. The results for individual models are given in Table 2, Table 3 and Table 4, where each table presents the results for one of the three optimization criteria used. The averages of sensitivity and specificity calculated based on the results of individual models for the first approach were 0.55 and 0.71, respectively, while for the second approach, the averages were 0.63 and 0.67.

Using the models developed by the first and second approach, consensus predictions were made for external validation set compounds. For the first approach, where the manually balanced training set was used, the obtained sensitivity and specificity for consensus predictions were 0.5 and 0.79, respectively. In the second approach, where models were developed by modified CPANN training algorithm, consensus predictions for external validation set resulted in sensitivity of 0.65 and specificity of 0.85. Comparing the consensus predictions with averages calculated from individual models, the consensus predictions based on the models from the second approach resulted in slightly increased sensitivity and significantly improved specificity. The consensus predictions were made considering and without considering the applicability domain of the models according to the method described by Minovski et al. [18]. In both cases, the consensus predictions were the same for all objects.

### 2.3. Descriptors

Since the composition of datasets and descriptors differed in the two approaches used for considering the imbalanced dataset, the importance of the selected descriptors was also assessed separately. In the first approach, where 24 models were selected, 143 unique descriptors were selected at least once, while in the second approach, 218 unique descriptors were used at least once. In order to assess the importance of selected descriptors, the following Equation (1) was used:

(1) $$I_{d}=\sum_{1}^{nm}\frac{\frac{s\times a}{n}}{nm}$$

In Equation (1), *nm* is the number of models where a descriptor could be potentially selected, *s* is 1 if descriptor is selected or 0 if it is not, *n* is the number of all of selected descriptors in the model, and *a* is the number of all descriptors that could be selected by a model (either 50, 98, or 181).

We removed descriptors that appeared less than four times in the accepted models for both approaches to lower the chances of a descriptor being randomly selected. Descriptors reaching I_d_ of at least 2 are given in Table 5 and Table 6 for the first and the second approach, respectively. To focus on the second modeling approach, which we assumed was superior, among the most important descriptors were two JGI descriptors (Table 6), which represented the total charge transfer (at topological distances 6 and 4). Interestingly, descriptor H%, which represented the percentage of H atoms in molecule, was placed highly in both tables. Another interpretable descriptor scoring high for the second approach was the Uc descriptor, which was correlated to the number of unsaturated bonds in a molecule.

It was difficult to draw conclusions of the role of the listed descriptors in hepatotoxicity. However, we plan to shed light on the effect of relevant descriptors in the future. To do so, we plan to improve the counter-propagation neural networks methodology to better discriminate compounds found in activity cliffs, which was a frequent event in the dataset used. Hopefully, future work will lead to a better understanding of the mechanisms of drug-induced liver injury.

## 3. Materials and Methods

### 3.1. Dataset

The dataset used in the study was compiled using data from five literature sources related to drug-induced liver injury (DILI) [12,21,22,23,24]. Names of the compounds in the literature sources were used to extract SMILES from PubChem [25]. From the collection of acquired data, chemical entities, such as proteins, larger peptides, inorganic compounds, compounds containing elements, which were very uncommon in the dataset, and mixtures of compounds were removed. We also removed compounds that are found in drugs where the only route of administration is topical according to DrugBank [26]. Compounds were, where applicable, protonated or deprotonated in Pipeline Pilot [27] to achieve canonical SMILES representation of molecules. Finally, duplicates with the same active pharmaceutical ingredient were removed, while their information for toxicity classes was retained. Three sources [21,23,24] tackled the DILI problem with more than two classes. We assigned compounds to two classes, DILI-positive and DILI-negative, according to the following rules. From source [21], most-DILI-concern and less-DILI-concern were considered DILI-positive and no-DILI-concern were considered DILI-negative. The toxicity classes “severe DILI” and ”non-severe DILI” from source [23] were considered DILI-positive, while the non-DILI class was considered DILI-negative. Compounds found in [24] were considered DILI-positive if they belonged to class A or B and DILI-negative if they belonged to class E. C, D, and E* classes from that source were not assigned to either the DILI-positive or DILI-negative class, and remained as such since they represent compounds with a lower likelihood of being toxic or nontoxic. In a way, such compounds represent unreliable data. Next, we checked if there were any conflicts in toxicity classes in the data from different sources. For example, if a compound was present in two out of five sources and classified as DILI-positive, we assigned the DILI-positive class to it. Similarly, if compound was present in three sources and classified as DILI-negative in two of them and as D in Livertox, the compound was not considered for further use (possible conflict of classes). From source [22], we extracted only compounds that had been manually checked for DILI (named calibration set in the source). In this way, we obtained 524 compounds that were used in our study.

### 3.2. Selection of Descriptors and Datasets

Descriptors used to represent compounds were 0-, 1-, and 2-dimensional descriptors calculated using Dragon [28] software. The selection of descriptors for modeling was made in two steps. The first step involved the removal of correlated and constant descriptors, and in the second step, the descriptors were reduced using Kohonen mapping. In the paper by Topliss and Edwards [29], where multiple regression models were studied, a limit of 0.8 for the pairwise correlation coefficient was suggested. However, we did not want to remove too many descriptors in the first step, thus we used a higher limit. The descriptors with pairwise correlation coefficient below 0.95 were kept. Descriptors with more than 70% constant values in the entire set were discarded. In that way, we obtained 580 descriptors, encoding a dataset of 524 compounds. Descriptors were normalized using z-scores. Compounds were mapped on 2-dimensional space using the Kohonen artificial neural network to select subsets used for the modeling. First, the objects for the external validation set were selected from the Kohonen top-map. We tried to maximize covered chemical space, while also attempting that validation set objects would reflect the density of all objects on the map, reflecting important aspects of diversity and closeness of sets [30]. Since we used two different approaches for dealing with an imbalanced dataset, each approach requiring a different size training set, we also used the validation sets of different sizes. After the exclusion of the external validation set, descriptors were again checked for constant values. Then, the number of descriptors was reduced. Kohonen maps of different sizes (5 × 5, 7 × 7, 10 × 10) were used to map the objects of the transposed data matrix. Kohonen top-maps were obtained, from which the closest and farthest objects (descriptor), considering the Euclidean distance to the neuron, were selected from each neuron. This resulted in sets containing 50, 98, and 181 descriptors used for modeling. The remaining compounds (excluding the validation set) were again mapped on the Kohonen top-map, this time using 50, 98, and 181 descriptors. The training set and two test sets (represented by TE1 and TE2) were selected from each top-map. Again, we tried to cover chemical space for each of the three sets, again taking the density of objects on the map into account to some degree. However, if a neuron was excited by up to two objects, they were favourably put in the training set. Descriptor values for all sets were normalized with z-score, with mean and standard deviation corresponding to the training set. Test set TE1 was used for model optimization; test set TE2 was used to estimate the performance of the models and select models, which were then externally validated using the external validation set.

**Figure 4:**
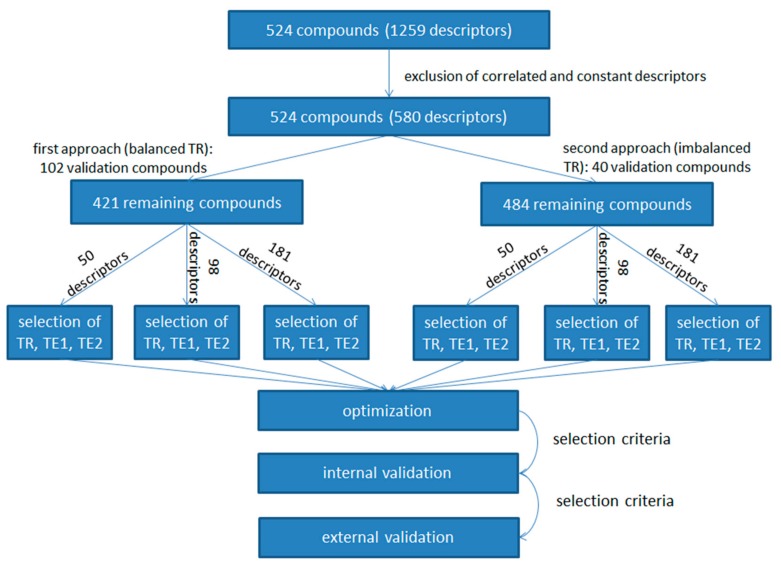
Modeling workflow. A total of 1259 Dragon descriptors were calculated for our dataset. The number of descriptors was reduced based on pairwise correlation and the frequency of the most common value. For the first modeling approach, where selection of the training set resulted in balanced classes, 102 validation objects were removed. For the second approach, where selection of the training set resulted in imbalanced classes, 40 objects were removed. For both approaches, the number of descriptors was further reduced: 50, 98, and 181 descriptors were selected. Based on the selected descriptors, objects for the training set (TR), test set 1 (TE1), and test set 2 (TE2) were selected. We applied the genetic algorithm for optimization of the models. Models passing the criteria were selected for internal validation with TE2. Lastly, models passing internal validation were externally validated with the corresponding validation set.

Information about the compounds and descriptors used are available in Supplementary Files “distribution_of_compounds_into_sets.xlsx” and “compound_descriptors.xlsx”. The workflow of the modeling approach used is schematically presented in Figure 4.

### 3.3. Models

When developing classification models, we used three different sets of descriptors, as explained in the previous Section 3.2. Due to the imbalanced nature of our dataset, having more non-toxic compounds than toxic, we also considered training with balanced training sets and an imbalanced training set. In that way, three different combinations of training and test sets were obtained for each of the two validation sets. The balanced training set contained 108 objects from each class, while the imbalanced training set was made up of 108 DILI-positive and 296 DILI-negative compounds. The imbalanced training set was further used in two ways. The first approach was to use all objects in the training set for each epoch of training. Secondly, due to the imbalanced nature of the training set, having 108 DILI-positive compounds and 296 DILI-negative compounds in the set, we used a balanced subset of 216 compounds in each epoch of training, which consisted of all 108 DILI-positive compounds and randomly selected 108 DILI-negative compounds for each epoch. Training was performed using the counter-propagation artificial neural network. The first approach of dealing with the imbalanced training set (i.e., not to deal with it) yielded no satisfactory results, so the first approach further in the text refers to the fixed balanced training set and the second approach refers to the imbalanced training set that was used as balanced in each epoch of training.

### 3.4. Optimization of Models Using Genetic Algorithm

We applied the genetic algorithm for the optimization of models. Optimizations were performed on test set TE1 or both the training and test set TE1 together, depending on the selected optimization criterion. We performed 7020 optimizations in total, each of them having a unique combination of an optimization criterion, a parameter describing the effect of number of selected descriptors on the cost function, number of neurons in the network, and the parameters used for genetic algorithm, such as the number of chromosomes for crossover. We selected three optimization criteria to optimize models. The selected optimization criteria (OC1, OC2, OC3) are written in Equations (2)–(4). The optimization criteria were based on the Matthew correlation coefficient (MCC) of test set TE1, the minimum of sensitivity and specificity of test set TE1, and the product of MCC of training and MCC of test set TE1. The effect of the number of selected descriptors (*Nselected*) on the optimization criterion was taken into account using Equation (5), where *p* is the parameter defined prior to each optimization and *Ndescriptors* is the total number of descriptors used in the training set. Matthew correlation coefficient, sensitivity, and specificity were calculated according to Equations (6)–(8), respectively. In Equations (6)–(8), *TP*, *TN*, *FP,* and *FN* indicate the number of true positive, true negative, false positive, and false negative predictions, respectively.

From all optimizations, we selected models where the sensitivity and specificity for all three sets (training, test TE1, and test TE2) were at least 0.7, and all three MCC were at least 0.5. Additionally, if any of the best three chromosomes during optimization had on average sensitivity and specificity of at least 0.7 for all three sets in the last 20 generations of optimization, all three models were also selected. Afterwards, we trained each such selected model 100 times using a random order of training objects. Models with average sensitivity and average specificity of 0.7 or higher for all three sets were finally selected as acceptable models.

OC1 = min(sensitivity(*test set TE1*),specificity (*test set TE1*))∙f, (2)

OC2= MCC(*test set* TE1)∙f, (3)

OC3= MCC(*training set*)∙MCC(*test set TE*1)∙f, (4)

f = 1 − *p*∙(*Nselected*-1)/*Ndescriptors*, (5)

MCC = (*TP*∙*TN – FP*∙*FN*)/(((*TP* + *FP*)∙(*TP* + *FN*)∙(*TN* + *FP*)∙(*TN* + *FN*))^1/2^), (6)

sensitivity = *TP*/(*TP* + *FN*), (7)

specificity = *TN*/(*TN* + *FP*). (8)

### 3.5. Kohonen and Counter-Propagation Artificial Neural Networks

Classification models were developed using counter-propagation artificial neural networks (CPANNs). Detailed descriptions of CPANNs can be found in the literature [19,20]. Here, a brief explanation of the training algorithm is given with a description of modifications made to the algorithm that was used when the number of objects in the two target classes of the training dataset was considerably different.

Counter-propagation artificial neural network models can be described as a 3D matrix of weights divided into two layers. A single column of weights in each layer represents one neuron. The upper layer of neurons in CPANNs is known as the Kohonen layer, and the layer beneath is the output layer, also known as the Grossberg layer. The weights in the Kohonen layer correspond to independent variables of objects (e.g., molecular descriptors), and the weights in the output layer correspond to dependent variables (e.g., target toxicity classes) of the input objects.

During the training, competitive learning is used in the Kohonen layer. The independent variables of an input object are compared to all the neurons in the Kohonen layer and the neuron that is the most similar to the object is selected as the central neuron, often called the “winning neuron”. The similarity between an object and a neuron is determined by calculating the Euclidean distance between the neuron weights and independent variables of the object. The neuron with the shortest Euclidean distance is selected as the central neuron. After the central neuron is determined, the corrections of the weights can be made. The corrections depend on a neighborhood function, learning rate, and differences between the current neuron weights and the corresponding variable of the input object. The neighborhood function defines the amount of correction made at a given topological distance from the central neuron. In our study, a triangular neighborhood function was used, where the correction was maximal on the central neuron and decreased with topological distance from the central neuron. The neighborhood function also decreased during the training so that at the end of the training only the weights corresponding to the central neuron were corrected. Learning rate, η(*t*), linearly decreased during the training according to Equation 9, with the largest value (*v*_max_) in the first iteration (*t* = 1) of the training and the smallest value (*v*_min_) at the end of the training (*t* = *t*_max_):

η(*t*) = *v*_min_ + (*v*_max_ – *v*_min_)∙(*t*_max_ – *t*)/(*t*_max_ – 1). (9)

The weights are corrected so that the weights in the Kohonen layer become more similar to the independent variables, and the weights in the output layer become more similar to the target values of the input object. The weights are updated according to Equation 10, where the second term in the sum represents the correction made at given iteration *t*:

^new^*w_i,j_* = ^old^*w*_i,j_ + η(*t*)∙a(*c*,*j,t*)∙(*x_i_* − ^old^*w_i,j_*). (10)

In Equation (10), ^new^*w_i,j_* and ^old^*w_i,j_* represent the new and previous values of the weight on neuron *j*, corresponding to variable *i* of the input object; *x_i_* represents the actual value of the variable *i* of the input object, and a(*c*,*j,t*) is the value of neighborhood function for neuron *j* and central neuron *c*. 

The training lasts for a predefined number of epochs, where one epoch means that each object from the training set is used exactly once for the training. Here, we introduced a modification that we made to the training procedure when the number of objects in one class was considerably larger than in the other class, i.e., when the imbalanced dataset was used for training. Normally, all the input objects are used in one epoch of training. Due to imbalanced data, we made a random subsampling of input objects without repetition prior to each epoch of training, so that the subsample contained a balanced number of objects from each class. Therefore, one epoch in such a case was equal to the number of training iterations when each object from the subsample was used exactly once. Practically, that meant that the neural network was trained with the number of objects equal to the number of objects in the subsample, and in each epoch a different training set was used. In this way, we still used all data from the training set, however the objects from the majority class were less frequently used during the training than those from the minority class.

### 3.6. Genetic Algorithm

The genetic algorithm was used for the optimization of the counter-propagation artificial neural network models. The genetic algorithm was employed for the selection of descriptors used in the model and for the optimization of learning rate parameters. A description of genetic algorithms is given in the literature [31]. Genetic algorithm optimization starts with the creation of an initial random population of chromosomes. Chromosomes may define the features to be used in a model and other parameters that may be involved in the development of the model. Using the encoded information from the chromosomes, models are built for each chromosome and ranked according to preselected optimization criterion. A new population of chromosomes is then created by mating chromosomes with good optimization criteria. Mutation operators are applied to the new population of chromosomes to introduce random genetic alterations in the chromosomes. The created population of chromosomes is again used for the creation of new models, and all the steps repeat until a predefined number of populations is reached or when no improvement in optimization criterion is observed.

## 4. Conclusions

Drug-induced liver injury (DILI) presents one of the major concerns in the drug development process. Using *in silico* approaches, one may efficiently evaluate the possible hepatotoxic effect of drugs. Imbalance of the dataset and activity cliffs present major obstacles to developing useful QSAR models. In this study, we encountered both difficulties when performing the modeling of DILI using counter-propagation artificial neural networks (CPANNs). The modification of the classical training algorithm with the integration of random subsampling within the training procedure of CPANN presented in this work was intended to solve the problem of dataset imbalance. A number of optimizations of CPANNs were performed using a genetic algorithm to find acceptable models. Based on the comparison of the results, we conclude that using an imbalanced set with balanced subsampling in each learning epoch is a better approach compared to using a fixed balanced set in the case of the counter-propagation artificial neural network learning methodology. The models obtained using the modified CPANN training algorithm were applied for consensus predictions and showed sensitivity of 0.65 and specificity of 0.85 for compounds in the external validation set.

## Figures and Tables

**Table 1:** Distribution of compounds into classes for the datasets used.

		TR		TE1		TE2		VA	
Ninit		Hepat.	Non-H.	Hepat.	Non-H.	Hepat.	Non-H.	Hepat.	Non-H.
182	a	108	296	20	20	20	20	20	20
	b	108	108	20	83	20	83	20	82
98	a	108	296	20	20	20	20	20	20
	b	108	108	20	83	20	83	20	82
50	a	108	296	20	20	20	20	20	20
	b	108	108	20	83	20	83	20	82

TR—training set; TE1—the first test set; TE2—the second test set; VA—external validation set. Ninit—Number of initial descriptors in the dataset. Hepat—Number of compounds in hepatotoxic class. Non-H—Number of compounds in non-hepatotoxic class. a— Imbalanced training set used during optimization. b—Manually balanced training set used during optimization.

**Table 2:** Average values of sensitivity and specificity obtained for models optimized using optimization criterion 1. Average values and standard deviations are given for 100 models built using selected descriptors and different permutations of objects in the training set.

OC1		TR		TE1		TE2		VA	
Nin.	model	Sens.	Spec.	Sens.	Spec.	Sens.	Spec.	Sens.	Spec.
182	1 ^a^	0.81 ± 0.02	0.87 ± 0.02	0.82 ± 0.07	0.84 ± 0.03	0.74 ± 0.07	0.70 ± 0.04	0.56 ± 0.06	0.68 ± 0.04
	2 ^b^	0.92 ± 0.02	0.79 ± 0.02	0.90 ± 0.06	0.92 ± 0.05	0.76 ± 0.08	0.78 ± 0.09	0.64 ± 0.08	0.72 ± 0.08
	3 ^b^	0.91 ± 0.02	0.79 ± 0.02	0.86 ± 0.06	0.90 ± 0.06	0.72 ± 0.09	0.78 ± 0.09	0.63 ± 0.08	0.73 ± 0.09
	4 ^b^	0.92 ± 0.02	0.78 ± 0.02	0.88 ± 0.07	0.90 ± 0.07	0.71 ± 0.07	0.77 ± 0.08	0.65 ± 0.07	0.75 ± 0.08
	5 ^b^	0.86 ± 0.03	0.76 ± 0.03	0.85 ± 0.07	0.86 ± 0.07	0.72 ± 0.08	0.71 ± 0.08	0.57 ± 0.08	0.78 ± 0.08
	6 ^b^	0.92 ± 0.02	0.81 ± 0.02	0.91 ± 0.06	0.92 ± 0.06	0.72 ± 0.07	0.74 ± 0.09	0.70 ± 0.07	0.66 ± 0.07
	7 ^b^	0.88 ± 0.03	0.77 ± 0.03	0.77 ± 0.08	0.83 ± 0.08	0.71 ± 0.07	0.71 ± 0.07	0.73 ± 0.07	0.77 ± 0.09
	8 ^b^	0.88 ± 0.02	0.77 ± 0.03	0.90 ± 0.04	0.88 ± 0.06	0.77 ± 0.08	0.70 ± 0.08	0.64 ± 0.07	0.68 ± 0.09
98	1 ^b^	0.89 ± 0.02	0.76 ± 0.02	0.91 ± 0.06	0.90 ± 0.05	0.72 ± 0.08	0.70 ± 0.09	0.74 ± 0.08	0.48 ± 0.10
	2 ^b^	0.89 ± 0.02	0.77 ± 0.02	0.87 ± 0.05	0.92 ± 0.04	0.77 ± 0.05	0.70 ± 0.06	0.60 ± 0.06	0.57 ± 0.08
	3 ^b^	0.89 ± 0.02	0.77 ± 0.02	0.86 ± 0.05	0.91 ± 0.04	0.75 ± 0.06	0.71 ± 0.07	0.60 ± 0.06	0.58 ± 0.09
	4 ^b^	0.90 ± 0.02	0.77 ± 0.02	0.86 ± 0.05	0.91 ± 0.06	0.76 ± 0.07	0.71 ± 0.06	0.68 ± 0.07	0.63 ± 0.08
	5 ^b^	0.94 ± 0.02	0.82 ± 0.02	0.84 ± 0.07	0.92 ± 0.05	0.70 ± 0.06	0.71 ± 0.07	0.69 ± 0.06	0.65 ± 0.07
	6 ^b^	0.85 ± 0.02	0.71 ± 0.02	0.88 ± 0.07	0.83 ± 0.07	0.73 ± 0.07	0.73 ± 0.07	0.69 ± 0.06	0.49 ± 0.09
	7 ^b^	0.95 ± 0.02	0.80 ± 0.02	0.88 ± 0.05	0.89 ± 0.07	0.75 ± 0.07	0.77 ± 0.08	0.54 ± 0.08	0.68 ± 0.07
	8 ^b^	0.96 ± 0.02	0.79 ± 0.02	0.87 ± 0.06	0.88 ± 0.06	0.75 ± 0.08	0.74 ± 0.08	0.53 ± 0.07	0.67 ± 0.07
	9 ^b^	0.95 ± 0.02	0.81 ± 0.02	0.87 ± 0.05	0.90 ± 0.06	0.76 ± 0.07	0.79 ± 0.07	0.53 ± 0.08	0.70 ± 0.07
	10 ^b^	0.78 ± 0.03	0.73 ± 0.03	0.81 ± 0.08	0.80 ± 0.06	0.71 ± 0.07	0.75 ± 0.06	0.63 ± 0.06	0.62 ± 0.07
	11 ^b^	0.87 ± 0.03	0.71 ± 0.03	0.80 ± 0.08	0.84 ± 0.07	0.72 ± 0.09	0.76 ± 0.08	0.64 ± 0.08	0.58 ± 0.09
	12 ^b^	0.93 ± 0.02	0.80 ± 0.02	0.93 ± 0.07	0.93 ± 0.05	0.71 ± 0.06	0.77 ± 0.08	0.66 ± 0.07	0.66 ± 0.08
	13 ^b^	0.82 ± 0.03	0.75 ± 0.02	0.84 ± 0.06	0.86 ± 0.08	0.72 ± 0.08	0.74 ± 0.07	0.72 ± 0.08	0.64 ± 0.06
	14 ^b^	0.85 ± 0.02	0.76 ± 0.02	0.86 ± 0.07	0.90 ± 0.07	0.70 ± 0.08	0.72 ± 0.08	0.70 ± 0.07	0.64 ± 0.07
	15 ^b^	0.79 ± 0.03	0.73 ± 0.02	0.90 ± 0.05	0.92 ± 0.06	0.77 ± 0.05	0.72 ± 0.07	0.64 ± 0.05	0.77 ± 0.07
	16 ^b^	0.79 ± 0.03	0.72 ± 0.02	0.89 ± 0.07	0.93 ± 0.05	0.80 ± 0.06	0.71 ± 0.06	0.64 ± 0.06	0.78 ± 0.07
50	1 ^a^	0.77 ± 0.02	0.71 ± 0.02	0.88 ± 0.04	0.83 ± 0.04	0.77 ± 0.04	0.73 ± 0.04	0.64 ± 0.03	0.68 ± 0.04
	2 ^a^	0.77 ± 0.02	0.72 ± 0.02	0.86 ± 0.04	0.84 ± 0.02	0.76 ± 0.04	0.75 ± 0.03	0.63 ± 0.04	0.71 ± 0.04
	3 ^a^	0.77 ± 0.02	0.71 ± 0.02	0.89 ± 0.04	0.82 ± 0.04	0.77 ± 0.03	0.73 ± 0.05	0.64 ± 0.03	0.68 ± 0.04
	4 ^a^	0.81 ± 0.02	0.79 ± 0.02	0.85 ± 0.08	0.83 ± 0.03	0.72 ± 0.06	0.71 ± 0.03	0.59 ± 0.07	0.73 ± 0.04
	5 ^a^	0.86 ± 0.03	0.86 ± 0.02	0.84 ± 0.06	0.81 ± 0.03	0.73 ± 0.08	0.71 ± 0.04	0.58 ± 0.08	0.70 ± 0.04
	6 ^b^	0.84 ± 0.03	0.72 ± 0.03	0.80 ± 0.07	0.84 ± 0.06	0.70 ± 0.07	0.71 ± 0.07	0.61 ± 0.08	0.64 ± 0.09

OC1—optimization criterion 1; TR—Training set; TE1—The first test set; TE2—The second test set; VA—External validation set. Nin.—Number of initial descriptors in the dataset; model—Model number; Sens.—Sensitivity; Spec.—Specificity. ^a^ Imbalanced training set used during optimization. ^b^ Manually balanced training set used during optimization.

**Table 3:** The average values of sensitivity and specificity obtained for models optimized using optimization criterion 2. Average values and standard deviations are given for 100 models built using selected descriptors and different permutations of objects in training set.

OC2		TR		TE1		TE2		VA	
Nin.	model	Sens.	Spec.	Sens.	Spec.	Sens.	Spec.	Sens.	Spec.
182	1 ^a^	0.92 ± 0.02	0.92 ± 0.02	0.93 ± 0.05	0.85 ± 0.03	0.76 ± 0.07	0.72 ± 0.04	0.39 ± 0.09	0.69 ± 0.04
	2 ^a^	0.93 ± 0.02	0.92 ± 0.02	0.94 ± 0.05	0.85 ± 0.04	0.76 ± 0.07	0.72 ± 0.04	0.39 ± 0.08	0.67 ± 0.04
	3 ^a^	0.90 ± 0.03	0.90 ± 0.03	0.83 ± 0.07	0.86 ± 0.04	0.72 ± 0.08	0.71 ± 0.04	0.50 ± 0.08	0.76 ± 0.04
	4 ^a^	0.89 ± 0.03	0.89 ± 0.03	0.84 ± 0.07	0.85 ± 0.04	0.74 ± 0.07	0.73 ± 0.04	0.50 ± 0.10	0.76 ± 0.04
	5 ^a^	0.77 ± 0.02	0.79 ± 0.03	0.82 ± 0.06	0.86 ± 0.04	0.70 ± 0.09	0.74 ± 0.04	0.61 ± 0.08	0.77 ± 0.04
	6 ^a^	0.80 ± 0.02	0.84 ± 0.02	0.91 ± 0.05	0.90 ± 0.04	0.73 ± 0.08	0.72 ± 0.04	0.58 ± 0.06	0.73 ± 0.03
	7 ^a^	0.81 ± 0.03	0.84 ± 0.02	0.93 ± 0.04	0.88 ± 0.04	0.71 ± 0.06	0.74 ± 0.04	0.56 ± 0.07	0.72 ± 0.03
	8 ^a^	0.82 ± 0.03	0.86 ± 0.02	0.84 ± 0.08	0.79 ± 0.04	0.73 ± 0.08	0.72 ± 0.04	0.57 ± 0.08	0.73 ± 0.04
	9 ^a^	0.82 ± 0.03	0.87 ± 0.02	0.86 ± 0.08	0.78 ± 0.04	0.74 ± 0.09	0.71 ± 0.04	0.59 ± 0.09	0.70 ± 0.04
	10 ^b^	0.92 ± 0.02	0.79 ± 0.02	0.85 ± 0.06	0.95 ± 0.05	0.72 ± 0.08	0.72 ± 0.08	0.65 ± 0.07	0.76 ± 0.08
	11 ^b^	0.92 ± 0.02	0.79 ± 0.02	0.86 ± 0.06	0.94 ± 0.05	0.71 ± 0.07	0.76 ± 0.07	0.68 ± 0.06	0.69 ± 0.10
	12 ^b^	0.92 ± 0.02	0.79 ± 0.02	0.85 ± 0.06	0.95 ± 0.05	0.72 ± 0.07	0.76 ± 0.07	0.67 ± 0.07	0.68 ± 0.07
	13 ^b^	0.91 ± 0.02	0.78 ± 0.02	0.83 ± 0.08	0.91 ± 0.06	0.76 ± 0.07	0.75 ± 0.08	0.63 ± 0.07	0.69 ± 0.08
	14 ^b^	0.92 ± 0.02	0.77 ± 0.02	0.84 ± 0.08	0.89 ± 0.06	0.73 ± 0.07	0.76 ± 0.09	0.62 ± 0.07	0.71 ± 0.08
	15 ^b^	0.95 ± 0.02	0.85 ± 0.02	0.91 ± 0.05	0.95 ± 0.05	0.70 ± 0.09	0.74 ± 0.08	0.67 ± 0.06	0.81 ± 0.07
	16 ^b^	0.95 ± 0.02	0.84 ± 0.02	0.92 ± 0.04	0.95 ± 0.05	0.72 ± 0.07	0.73 ± 0.07	0.69 ± 0.05	0.81 ± 0.07
	17 ^b^	0.95 ± 0.02	0.84 ± 0.02	0.92 ± 0.04	0.95 ± 0.05	0.73 ± 0.09	0.75 ± 0.08	0.68 ± 0.06	0.80 ± 0.07
	18 ^b^	0.88 ± 0.02	0.75 ± 0.02	0.85 ± 0.07	0.92 ± 0.06	0.72 ± 0.07	0.72 ± 0.07	0.61 ± 0.10	0.66 ± 0.08
	19 ^b^	0.90 ± 0.02	0.78 ± 0.02	0.84 ± 0.07	0.82 ± 0.07	0.74 ± 0.08	0.71 ± 0.08	0.59 ± 0.08	0.63 ± 0.09
	20 ^b^	0.90 ± 0.02	0.77 ± 0.02	0.80 ± 0.09	0.81 ± 0.09	0.75 ± 0.06	0.72 ± 0.09	0.71 ± 0.09	0.64 ± 0.09
	21 ^b^	0.91 ± 0.02	0.79 ± 0.02	0.79 ± 0.08	0.81 ± 0.07	0.73 ± 0.05	0.72 ± 0.07	0.60 ± 0.06	0.60 ± 0.08
98	1 ^b^	0.82 ± 0.03	0.75 ± 0.02	0.86 ± 0.08	0.82 ± 0.08	0.73 ± 0.07	0.77 ± 0.08	0.70 ± 0.07	0.64 ± 0.09
	2 ^b^	0.91 ± 0.02	0.78 ± 0.02	0.78 ± 0.08	0.91 ± 0.06	0.71 ± 0.08	0.71 ± 0.08	0.61 ± 0.08	0.74 ± 0.08
	3 ^b^	0.89 ± 0.02	0.76 ± 0.02	0.86 ± 0.08	0.91 ± 0.06	0.70 ± 0.07	0.74 ± 0.06	0.64 ± 0.07	0.59 ± 0.08
	4 ^b^	0.89 ± 0.03	0.73 ± 0.02	0.89 ± 0.07	0.92 ± 0.05	0.73 ± 0.07	0.72 ± 0.08	0.67 ± 0.08	0.56 ± 0.09
	5 ^b^	0.85 ± 0.02	0.78 ± 0.02	0.84 ± 0.06	0.93 ± 0.06	0.77 ± 0.06	0.79 ± 0.10	0.63 ± 0.08	0.74 ± 0.06
	6 ^b^	0.88 ± 0.03	0.77 ± 0.03	0.78 ± 0.08	0.81 ± 0.09	0.75 ± 0.07	0.79 ± 0.07	0.70 ± 0.09	0.71 ± 0.06
	7 ^b^	0.87 ± 0.02	0.78 ± 0.02	0.78 ± 0.07	0.90 ± 0.08	0.76 ± 0.06	0.84 ± 0.07	0.62 ± 0.07	0.73 ± 0.07
	8 ^b^	0.93 ± 0.02	0.76 ± 0.02	0.86 ± 0.07	0.93 ± 0.05	0.81 ± 0.07	0.72 ± 0.08	0.68 ± 0.06	0.66 ± 0.06
	9 ^b^	0.91 ± 0.02	0.76 ± 0.02	0.83 ± 0.08	0.91 ± 0.06	0.79 ± 0.06	0.75 ± 0.07	0.60 ± 0.06	0.68 ± 0.08
	10 ^b^	0.91 ± 0.02	0.76 ± 0.02	0.80 ± 0.08	0.90 ± 0.06	0.78 ± 0.07	0.75 ± 0.07	0.60 ± 0.08	0.68 ± 0.07
	11 ^b^	0.91 ± 0.02	0.75 ± 0.02	0.81 ± 0.08	0.91 ± 0.06	0.77 ± 0.07	0.75 ± 0.08	0.60 ± 0.08	0.67 ± 0.09
	12 ^b^	0.82 ± 0.03	0.70 ± 0.03	0.84 ± 0.08	0.85 ± 0.07	0.75 ± 0.08	0.76 ± 0.08	0.68 ± 0.09	0.59 ± 0.07
	13 ^b^	0.86 ± 0.03	0.74 ± 0.03	0.83 ± 0.08	0.88 ± 0.06	0.70 ± 0.07	0.71 ± 0.07	0.55 ± 0.08	0.71 ± 0.08
	14 ^b^	0.91 ± 0.02	0.78 ± 0.02	0.80 ± 0.08	0.90 ± 0.05	0.70 ± 0.08	0.82 ± 0.06	0.67 ± 0.07	0.55 ± 0.08
	15 ^b^	0.91 ± 0.02	0.77 ± 0.02	0.81 ± 0.08	0.89 ± 0.06	0.71 ± 0.06	0.81 ± 0.06	0.68 ± 0.08	0.57 ± 0.08
	16 ^b^	0.86 ± 0.02	0.78 ± 0.02	0.88 ± 0.07	0.93 ± 0.06	0.73 ± 0.08	0.75 ± 0.06	0.64 ± 0.06	0.73 ± 0.07
	17 ^b^	0.85 ± 0.03	0.79 ± 0.02	0.85 ± 0.08	0.92 ± 0.06	0.72 ± 0.08	0.79 ± 0.05	0.64 ± 0.06	0.74 ± 0.07
	18 ^b^	0.81 ± 0.02	0.79 ± 0.02	0.87 ± 0.06	0.92 ± 0.05	0.76 ± 0.06	0.81 ± 0.06	0.65 ± 0.06	0.75 ± 0.07
	19 ^b^	0.91 ± 0.02	0.77 ± 0.02	0.89 ± 0.07	0.88 ± 0.07	0.73 ± 0.09	0.71 ± 0.09	0.61 ± 0.08	0.51 ± 0.09
	20 ^b^	0.87 ± 0.02	0.73 ± 0.02	0.78 ± 0.08	0.83 ± 0.06	0.73 ± 0.08	0.73 ± 0.07	0.64 ± 0.07	0.56 ± 0.08
	21 ^b^	0.90 ± 0.02	0.72 ± 0.02	0.81 ± 0.07	0.88 ± 0.06	0.71 ± 0.07	0.72 ± 0.08	0.53 ± 0.08	0.63 ± 0.09
50	1 ^a^	0.76 ± 0.03	0.74 ± 0.03	0.85 ± 0.07	0.78 ± 0.04	0.71 ± 0.08	0.72 ± 0.05	0.61 ± 0.08	0.70 ± 0.04
	2 ^a^	0.77 ± 0.03	0.74 ± 0.04	0.86 ± 0.07	0.78 ± 0.05	0.72 ± 0.07	0.71 ± 0.04	0.61 ± 0.07	0.69 ± 0.04
	3 ^a^	0.77 ± 0.04	0.74 ± 0.04	0.85 ± 0.08	0.78 ± 0.04	0.71 ± 0.08	0.71 ± 0.05	0.60 ± 0.07	0.69 ± 0.04
	4 ^b^	0.97 ± 0.01	0.83 ± 0.02	0.88 ± 0.06	0.85 ± 0.08	0.71 ± 0.08	0.72 ± 0.08	0.55 ± 0.07	0.79 ± 0.08
	5 ^b^	0.96 ± 0.02	0.83 ± 0.02	0.87 ± 0.07	0.85 ± 0.06	0.71 ± 0.08	0.70 ± 0.08	0.59 ± 0.07	0.75 ± 0.08

OC2—optimization criterion 2; TR—Training set; TE1—The first test set; TE2—The second test set; VA—External validation set. Nin.—Number of initial descriptors in the dataset; model—Model number; Sens.—Sensitivity; Spec.—Specificity. ^a^ Imbalanced training set used during optimization. ^b^ Manually balanced training set used during optimization.

**Table 4:** The average values of sensitivity and specificity obtained for models optimized using optimization criterion 3. Average values and standard deviations are given for 100 models built using selected descriptors and different permutations of objects in training set.

OC3		TR		TE1		TE2		VA	
Nin.	model	Sens.	Spec.	Sens.	Spec.	Sens.	Spec.	Sens.	Spec.
182	1 ^a^	0.91 ± 0.03	0.91 ± 0.03	0.88 ± 0.06	0.81 ± 0.04	0.71 ± 0.08	0.70 ± 0.04	0.47 ± 0.08	0.74 ± 0.04
	2 ^a^	0.90 ± 0.03	0.90 ± 0.03	0.83 ± 0.06	0.86 ± 0.04	0.70 ± 0.09	0.71 ± 0.04	0.52 ± 0.09	0.74 ± 0.04
	3 ^a^	0.86 ± 0.03	0.85 ± 0.03	0.86 ± 0.06	0.83 ± 0.05	0.77 ± 0.08	0.70 ± 0.04	0.50 ± 0.10	0.73 ± 0.04
	4 ^b^	0.94 ± 0.02	0.82 ± 0.02	0.93 ± 0.06	0.89 ± 0.06	0.74 ± 0.07	0.72 ± 0.08	0.63 ± 0.08	0.65 ± 0.07
	5 ^b^	0.94 ± 0.02	0.81 ± 0.02	0.94 ± 0.05	0.91 ± 0.06	0.73 ± 0.08	0.71 ± 0.08	0.65 ± 0.07	0.64 ± 0.07
	6 ^b^	0.93 ± 0.02	0.84 ± 0.02	0.90 ± 0.04	0.96 ± 0.05	0.72 ± 0.07	0.71 ± 0.09	0.69 ± 0.06	0.66 ± 0.09
	7 ^b^	0.93 ± 0.02	0.83 ± 0.02	0.91 ± 0.04	0.96 ± 0.05	0.73 ± 0.07	0.71 ± 0.08	0.66 ± 0.07	0.66 ± 0.08
	8 ^b^	0.93 ± 0.02	0.82 ± 0.02	0.90 ± 0.05	0.92 ± 0.06	0.73 ± 0.08	0.71 ± 0.07	0.61 ± 0.06	0.74 ± 0.07
	9 ^b^	0.93 ± 0.02	0.82 ± 0.02	0.89 ± 0.05	0.92 ± 0.06	0.76 ± 0.07	0.73 ± 0.07	0.63 ± 0.07	0.75 ± 0.07
	10 ^b^	0.89 ± 0.03	0.77 ± 0.03	0.84 ± 0.06	0.90 ± 0.07	0.75 ± 0.08	0.72 ± 0.09	0.73 ± 0.07	0.64 ± 0.07
	11 ^b^	0.88 ± 0.02	0.78 ± 0.02	0.89 ± 0.07	0.91 ± 0.06	0.70 ± 0.08	0.72 ± 0.09	0.64 ± 0.08	0.68 ± 0.08
	12 ^b^	0.86 ± 0.02	0.81 ± 0.03	0.88 ± 0.08	0.96 ± 0.06	0.75 ± 0.07	0.73 ± 0.08	0.65 ± 0.07	0.72 ± 0.07
	13 ^b^	0.87 ± 0.02	0.81 ± 0.02	0.86 ± 0.07	0.95 ± 0.05	0.73 ± 0.07	0.72 ± 0.09	0.68 ± 0.06	0.72 ± 0.08
	14 ^b^	0.87 ± 0.02	0.80 ± 0.02	0.87 ± 0.07	0.95 ± 0.05	0.75 ± 0.08	0.72 ± 0.08	0.67 ± 0.07	0.73 ± 0.07
	15 ^b^	0.92 ± 0.02	0.83 ± 0.02	0.84 ± 0.06	0.92 ± 0.06	0.72 ± 0.07	0.71 ± 0.08	0.63 ± 0.06	0.73 ± 0.06
	16 ^b^	0.93 ± 0.02	0.83 ± 0.02	0.85 ± 0.07	0.91 ± 0.06	0.72 ± 0.07	0.71 ± 0.09	0.64 ± 0.06	0.78 ± 0.08
	17 ^b^	0.94 ± 0.02	0.81 ± 0.02	0.93 ± 0.06	0.91 ± 0.06	0.75 ± 0.07	0.77 ± 0.08	0.66 ± 0.06	0.72 ± 0.08
	18 ^b^	0.94 ± 0.02	0.81 ± 0.02	0.92 ± 0.06	0.91 ± 0.07	0.74 ± 0.08	0.72 ± 0.08	0.65 ± 0.05	0.72 ± 0.08
	19 ^b^	0.96 ± 0.01	0.86 ± 0.02	0.92 ± 0.06	0.95 ± 0.05	0.70 ± 0.07	0.70 ± 0.06	0.58 ± 0.06	0.72 ± 0.06
98	1 ^a^	0.86 ± 0.03	0.89 ± 0.02	0.91 ± 0.06	0.76 ± 0.04	0.71 ± 0.08	0.71 ± 0.05	0.52 ± 0.10	0.65 ± 0.04
	2 ^b^	0.95 ± 0.02	0.82 ± 0.02	0.79 ± 0.07	0.93 ± 0.06	0.70 ± 0.06	0.77 ± 0.06	0.57 ± 0.07	0.68 ± 0.06
	3 ^b^	0.91 ± 0.02	0.78 ± 0.02	0.82 ± 0.07	0.88 ± 0.06	0.70 ± 0.08	0.71 ± 0.07	0.68 ± 0.08	0.61 ± 0.08
	4 ^b^	0.91 ± 0.02	0.79 ± 0.02	0.80 ± 0.07	0.90 ± 0.05	0.73 ± 0.07	0.71 ± 0.06	0.69 ± 0.07	0.64 ± 0.07
	5 ^b^	0.92 ± 0.02	0.80 ± 0.02	0.85 ± 0.07	0.95 ± 0.05	0.71 ± 0.09	0.74 ± 0.07	0.63 ± 0.07	0.65 ± 0.07
	6 ^b^	0.87 ± 0.02	0.77 ± 0.02	0.78 ± 0.08	0.87 ± 0.07	0.73 ± 0.06	0.72 ± 0.07	0.63 ± 0.07	0.63 ± 0.07
	7 ^b^	0.86 ± 0.02	0.75 ± 0.02	0.79 ± 0.07	0.87 ± 0.07	0.73 ± 0.07	0.71 ± 0.07	0.62 ± 0.07	0.63 ± 0.08
	8 ^b^	0.87 ± 0.02	0.76 ± 0.02	0.79 ± 0.08	0.85 ± 0.08	0.73 ± 0.06	0.73 ± 0.08	0.60 ± 0.08	0.61 ± 0.09
	9 ^b^	0.86 ± 0.03	0.75 ± 0.03	0.73 ± 0.09	0.92 ± 0.06	0.72 ± 0.07	0.72 ± 0.08	0.67 ± 0.09	0.64 ± 0.08
	10 ^b^	0.87 ± 0.02	0.77 ± 0.02	0.74 ± 0.09	0.93 ± 0.06	0.75 ± 0.06	0.75 ± 0.06	0.70 ± 0.08	0.58 ± 0.09
	11 ^b^	0.92 ± 0.02	0.80 ± 0.02	0.84 ± 0.07	0.91 ± 0.06	0.76 ± 0.08	0.80 ± 0.06	0.71 ± 0.07	0.68 ± 0.07
	12 ^b^	0.92 ± 0.02	0.80 ± 0.02	0.84 ± 0.06	0.90 ± 0.06	0.71 ± 0.07	0.80 ± 0.08	0.66 ± 0.08	0.68 ± 0.08
	13 ^b^	0.92 ± 0.02	0.79 ± 0.02	0.86 ± 0.07	0.93 ± 0.05	0.73 ± 0.08	0.80 ± 0.08	0.68 ± 0.07	0.68 ± 0.07
	14 ^b^	0.86 ± 0.02	0.79 ± 0.03	0.74 ± 0.06	0.92 ± 0.06	0.71 ± 0.08	0.78 ± 0.08	0.58 ± 0.07	0.73 ± 0.09
	15 ^b^	0.86 ± 0.03	0.79 ± 0.03	0.72 ± 0.07	0.91 ± 0.05	0.72 ± 0.08	0.78 ± 0.08	0.53 ± 0.07	0.68 ± 0.09
	16 ^b^	0.92 ± 0.02	0.79 ± 0.02	0.88 ± 0.06	0.90 ± 0.05	0.75 ± 0.05	0.72 ± 0.07	0.64 ± 0.06	0.64 ± 0.08
	17 ^b^	0.93 ± 0.02	0.80 ± 0.02	0.73 ± 0.09	0.96 ± 0.04	0.70 ± 0.07	0.71 ± 0.08	0.64 ± 0.07	0.70 ± 0.08
	18 ^b^	0.93 ± 0.02	0.80 ± 0.02	0.79 ± 0.08	0.93 ± 0.05	0.72 ± 0.07	0.77 ± 0.08	0.63 ± 0.07	0.68 ± 0.08
	19 ^b^	0.92 ± 0.02	0.81 ± 0.02	0.74 ± 0.08	0.87 ± 0.06	0.73 ± 0.08	0.79 ± 0.06	0.67 ± 0.09	0.65 ± 0.08
	20 ^b^	0.91 ± 0.02	0.81 ± 0.02	0.83 ± 0.07	0.91 ± 0.05	0.74 ± 0.07	0.76 ± 0.07	0.56 ± 0.08	0.68 ± 0.08
	21 ^b^	0.91 ± 0.02	0.81 ± 0.02	0.83 ± 0.08	0.92 ± 0.06	0.74 ± 0.07	0.75 ± 0.08	0.57 ± 0.09	0.66 ± 0.08
	22 ^b^	0.92 ± 0.02	0.81 ± 0.02	0.85 ± 0.07	0.95 ± 0.05	0.76 ± 0.07	0.81 ± 0.07	0.60 ± 0.08	0.68 ± 0.09
	23 ^b^	0.91 ± 0.02	0.80 ± 0.02	0.77 ± 0.08	0.87 ± 0.07	0.72 ± 0.08	0.70 ± 0.07	0.63 ± 0.07	0.65 ± 0.08
	24 ^b^	0.91 ± 0.02	0.80 ± 0.03	0.78 ± 0.09	0.89 ± 0.05	0.72 ± 0.08	0.73 ± 0.07	0.59 ± 0.07	0.64 ± 0.07
	25 ^b^	0.91 ± 0.02	0.81 ± 0.02	0.84 ± 0.08	0.96 ± 0.04	0.71 ± 0.07	0.74 ± 0.07	0.58 ± 0.07	0.69 ± 0.09
	26 ^b^	0.94 ± 0.02	0.84 ± 0.02	0.78 ± 0.08	0.92 ± 0.06	0.83 ± 0.06	0.72 ± 0.08	0.62 ± 0.08	0.66 ± 0.07
	27 ^b^	0.94 ± 0.02	0.84 ± 0.02	0.81 ± 0.09	0.93 ± 0.06	0.83 ± 0.06	0.74 ± 0.07	0.59 ± 0.08	0.66 ± 0.06
	28 ^b^	0.94 ± 0.02	0.84 ± 0.02	0.81 ± 0.07	0.92 ± 0.06	0.83 ± 0.05	0.72 ± 0.09	0.61 ± 0.08	0.63 ± 0.06
	29 ^b^	0.96 ± 0.02	0.85 ± 0.02	0.83 ± 0.08	0.95 ± 0.05	0.70 ± 0.09	0.74 ± 0.08	0.63 ± 0.10	0.63 ± 0.09
	30 ^b^	0.96 ± 0.01	0.85 ± 0.02	0.83 ± 0.07	0.92 ± 0.06	0.72 ± 0.07	0.70 ± 0.07	0.54 ± 0.07	0.66 ± 0.08
	31 ^b^	0.97 ± 0.02	0.86 ± 0.02	0.82 ± 0.07	0.94 ± 0.04	0.72 ± 0.07	0.72 ± 0.07	0.54 ± 0.07	0.67 ± 0.07
	32 ^b^	0.96 ± 0.02	0.85 ± 0.02	0.85 ± 0.07	0.95 ± 0.04	0.71 ± 0.08	0.71 ± 0.06	0.55 ± 0.07	0.62 ± 0.07
	33 ^b^	0.88 ± 0.03	0.77 ± 0.03	0.82 ± 0.08	0.91 ± 0.07	0.78 ± 0.07	0.73 ± 0.08	0.67 ± 0.08	0.68 ± 0.08
	34 ^b^	0.88 ± 0.03	0.77 ± 0.03	0.80 ± 0.08	0.91 ± 0.06	0.77 ± 0.07	0.73 ± 0.07	0.66 ± 0.08	0.70 ± 0.08
	35 ^b^	0.88 ± 0.03	0.77 ± 0.03	0.80 ± 0.07	0.91 ± 0.06	0.75 ± 0.08	0.72 ± 0.06	0.66 ± 0.09	0.68 ± 0.08
	36 ^b^	0.96 ± 0.02	0.83 ± 0.02	0.74 ± 0.07	0.93 ± 0.06	0.79 ± 0.06	0.71 ± 0.06	0.65 ± 0.07	0.66 ± 0.07
	37 ^b^	0.95 ± 0.02	0.82 ± 0.02	0.73 ± 0.07	0.94 ± 0.05	0.79 ± 0.06	0.71 ± 0.06	0.65 ± 0.06	0.65 ± 0.07
	38 ^b^	0.96 ± 0.02	0.82 ± 0.02	0.76 ± 0.07	0.90 ± 0.06	0.78 ± 0.06	0.70 ± 0.07	0.65 ± 0.08	0.65 ± 0.06
	39 ^b^	0.86 ± 0.03	0.71 ± 0.03	0.85 ± 0.08	0.83 ± 0.06	0.70 ± 0.09	0.72 ± 0.08	0.68 ± 0.07	0.63 ± 0.09
	40 ^b^	0.87 ± 0.02	0.71 ± 0.03	0.82 ± 0.08	0.82 ± 0.06	0.74 ± 0.08	0.71 ± 0.08	0.66 ± 0.06	0.61 ± 0.08
	41 ^b^	0.83 ± 0.02	0.77 ± 0.02	0.80 ± 0.08	0.90 ± 0.06	0.71 ± 0.07	0.78 ± 0.06	0.54 ± 0.07	0.59 ± 0.08
	42 ^b^	0.82 ± 0.02	0.76 ± 0.03	0.80 ± 0.07	0.90 ± 0.06	0.76 ± 0.08	0.73 ± 0.07	0.53 ± 0.07	0.54 ± 0.06
	43 ^b^	0.83 ± 0.03	0.74 ± 0.02	0.81 ± 0.08	0.91 ± 0.05	0.76 ± 0.08	0.73 ± 0.06	0.56 ± 0.07	0.54 ± 0.07
	44 ^b^	0.95 ± 0.02	0.79 ± 0.02	0.71 ± 0.09	0.90 ± 0.04	0.74 ± 0.07	0.71 ± 0.08	0.64 ± 0.07	0.69 ± 0.07
	45 ^b^	0.94 ± 0.02	0.81 ± 0.02	0.84 ± 0.06	0.90 ± 0.06	0.71 ± 0.07	0.79 ± 0.06	0.76 ± 0.07	0.65 ± 0.07
50	1 ^a^	0.92 ± 0.02	0.92 ± 0.02	0.82 ± 0.05	0.79 ± 0.04	0.72 ± 0.07	0.70 ± 0.03	0.53 ± 0.06	0.69 ± 0.04
	2 ^a^	0.91 ± 0.02	0.93 ± 0.02	0.83 ± 0.07	0.83 ± 0.04	0.72 ± 0.07	0.70 ± 0.04	0.60 ± 0.07	0.66 ± 0.04
	3 ^b^	0.92 ± 0.02	0.78 ± 0.02	0.82 ± 0.07	0.83 ± 0.07	0.71 ± 0.09	0.71 ± 0.06	0.61 ± 0.07	0.77 ± 0.08
	4 ^b^	0.87 ± 0.03	0.76 ± 0.02	0.84 ± 0.09	0.85 ± 0.07	0.73 ± 0.08	0.71 ± 0.07	0.66 ± 0.07	0.66 ± 0.08
	5 ^b^	0.87 ± 0.03	0.75 ± 0.02	0.85 ± 0.07	0.83 ± 0.09	0.72 ± 0.08	0.72 ± 0.08	0.66 ± 0.07	0.66 ± 0.07
	6 ^b^	0.95 ± 0.02	0.83 ± 0.02	0.80 ± 0.08	0.93 ± 0.06	0.72 ± 0.07	0.73 ± 0.07	0.55 ± 0.08	0.62 ± 0.07
	7 ^b^	0.96 ± 0.02	0.83 ± 0.02	0.81 ± 0.07	0.93 ± 0.06	0.71 ± 0.08	0.74 ± 0.07	0.58 ± 0.07	0.63 ± 0.07

OC3—optimization criterion 3; TR—Training set; TE1—The first test set; TE2—The second test set; VA—External validation set. Nin.—Number of initial descriptors in the dataset; model—Model number; Sens.—Sensitivity; Spec.—Specificity. ^a^ Imbalanced training set used during optimization. ^b^ Manually balanced training set used during optimization.

**Table 5:** Most important descriptors for models using the first modeling approach.

Descriptor	Description	I_d_
J_D/Dt	Balaban-like index from distance/detour matrix	8.945987
GATS5v	Geary autocorrelation of lag 5 weighted by van der Waals volume	8.042931
H%	Percentage of H atoms	7.579833
SpMin1_Bh(s)	Smallest eigenvalue n. 1 of Burden matrix weighted by I-state	6.506972
CATS2D_02_AA	CATS2D Acceptor-Acceptor at lag 02	5.406386
IC2	Information content index (neighborhood symmetry of 2-order)	5.3672
GATS1v	Geary autocorrelation of lag 1 weighted by van der Waals volume	4.810587
GATS2v	Geary autocorrelation of lag 2 weighted by van der Waals volume	4.727913
BAC	Balaban centric index	4.58365
SpPosA_X	Normalized spectral positive sum from chi matrix	4.303807
P_VSA_LogP_6	P_VSA-like on LogP, bin 6	3.877732
C-006	CH2RX	3.640303
P_VSA_e_3	P_VSA-like on Sanderson electronegativity, bin 3	3.475949
P_VSA_MR_2	P_VSA-like on Molar Refractivity, bin 2	3.236547
MATS8m	Moran autocorrelation of lag 8 weighted by mass	3.138591
nCsp3	Number of sp3 hybridized carbon atoms	2.675997
PDI	Packing density index	2.585321
P_VSA_m_4	P_VSA-like on mass, bin 4	2.511289
SpAD_EA(dm)	Spectral absolute deviation from edge adjacency mat. weighted by dipole moment	2.35969
CATS2D_04_AA	CATS2D Acceptor-Acceptor at lag 04	2.332174
X5Av	Average valence connectivity index of order 5	2.196837
X5A	Average connectivity index of order 5	2.100552

**Table 6:** Most important descriptors for models using the second modeling approach.

Descriptor	Description	I_d_
JGI6	Mean topological charge index of order 6	3.502671
JGI4	Mean topological charge index of order 4	3.398279
SdssC	Sum of dssC E-states	3.372717
H%	Percentage of H atoms	3.287295
Uc	Unsaturation count	3.071672
P_VSA_LogP_6	P_VSA-like on LogP, bin 6	2.985918
H-052	H attached to C0(sp3) with 1X attached to next C	2.805648
MAXDN	Maximal electrotopological negative variation	2.620215
Chi1_EA(dm)	Connectivity-like index of order 1 from edge adjacency mat. Weighted by dipole moment	2.576782
SpMax_B(m)	Leading eigenvalue from Burden matrix weighted by mass	2.540987
GATS5m	Geary autocorrelation of lag 5 weighted by mass	2.480224
SpAD_EA(dm)	Spectral absolute deviation from edge adjacency mat. Weighted by Dipole moment	2.416726
GATS1i	Geary autocorrelation of lag 1 weighted by ionization potential	2.365205
SsssN	Sum of sssN E-states	2.352358
SpMAD_EA(bo)	Spectral mean absolute deviation from edge adjacency mat. Weighted by bond order	2.344266
ChiA_B(s)	Average Randic-like index from Burden matrix weighted by I-State	2.338868
NssO	Number of atoms of type ssO	2.223777
VE2sign_A	Average coefficient of the last eigenvector from adjacency matrix	2.169277
MATS2p	Moran autocorrelation of lag 2 weighted by polarizability	2.169277
MATS1p	Moran autocorrelation of lag 1 weighted by polarizability	2.103962
SpMin1_Bh(v)	Smallest eigenvalue n. 1 of Burden matrix weighted by van der Waals volume	2.089443
ChiA_B(v)	Average Randic-like index from Burden matrix weighted by van der Waals volume	2.043667
Rbrid	Ring bridge count	2.040469
nCsp3	Number of sp3 hybridized Carbon atoms	2.038696
C-040	R-C(=X)-X / R-C#X / X=C=X	2.022

## Supplementary Materials

The following are available online, Supplementary1.pdf: Results of Principal Component Analysis, compound_descriptors.xlsx, distribution_of_compounds_into_sets.xlsx.
