# Supplementary material for: Hepatotoxicity Modeling Using Counter-Propagation Artificial Neural Networks: Handling an Imbalanced Classification Problem
Source: Molecules. 2020 Jan 23;25(3):481. doi: 10.3390/molecules25030481 (PMC7037161; doi:10.3390/molecules25030481)
Supplement: Supplementary file 1 [file molecules-25-00481-s001.zip › molecules-697870-supplement/Supplementary1.pdf]

Results of Principal Component Analysis

Distribution of compounds belonging to external validation set on Principal Component Analysis (PCA) score plots is shown in Figure S1 and Figure S2 where red color indicates validation set compounds. Further analysis with PCA was made to assess the possibilities of separating hepatotoxic and non-hepatotoxic drugs with the first few principal components, however no satisfactory separation of compounds in hepatotoxic and non-hepatotoxic class could be achieved. The results are shown in Figure S3 and Figure S4 for the first and the second approach, respectively, where external validation set was removed prior the PCA analysis. As can be observed from Figures S3 and S4, approximately 37% of variance could be explained using the first two principal components (PC1 and PC2) and compounds belonging to different classes could not be separated by using PCA.

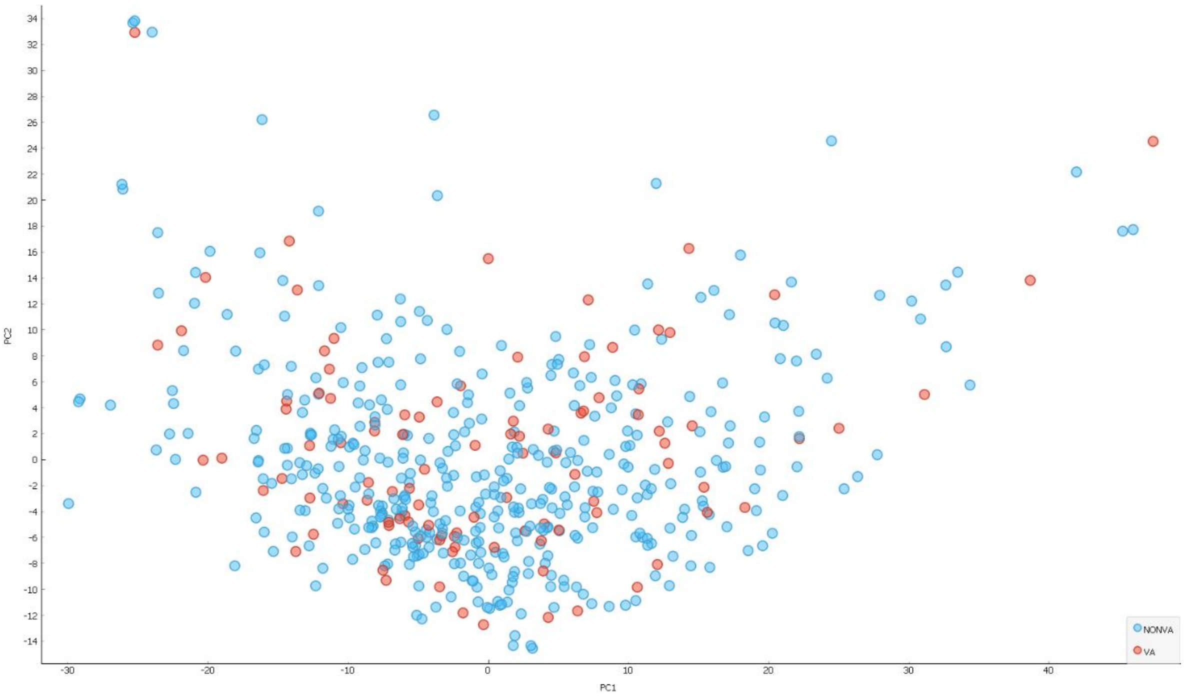

Figure S1: Principal component analysis (PCA) of dataset. Graph depicts arrangement of objects in multivariable space along the two axes with the largest variance. Principal component 1 (PC1) and principal component 2 (PC2) cover 37% of variance in the dataset. Red dots depict 102 objects in the external validation set (VA) used in the first approach, blue dots depict 422 remaining objects (NONVA).

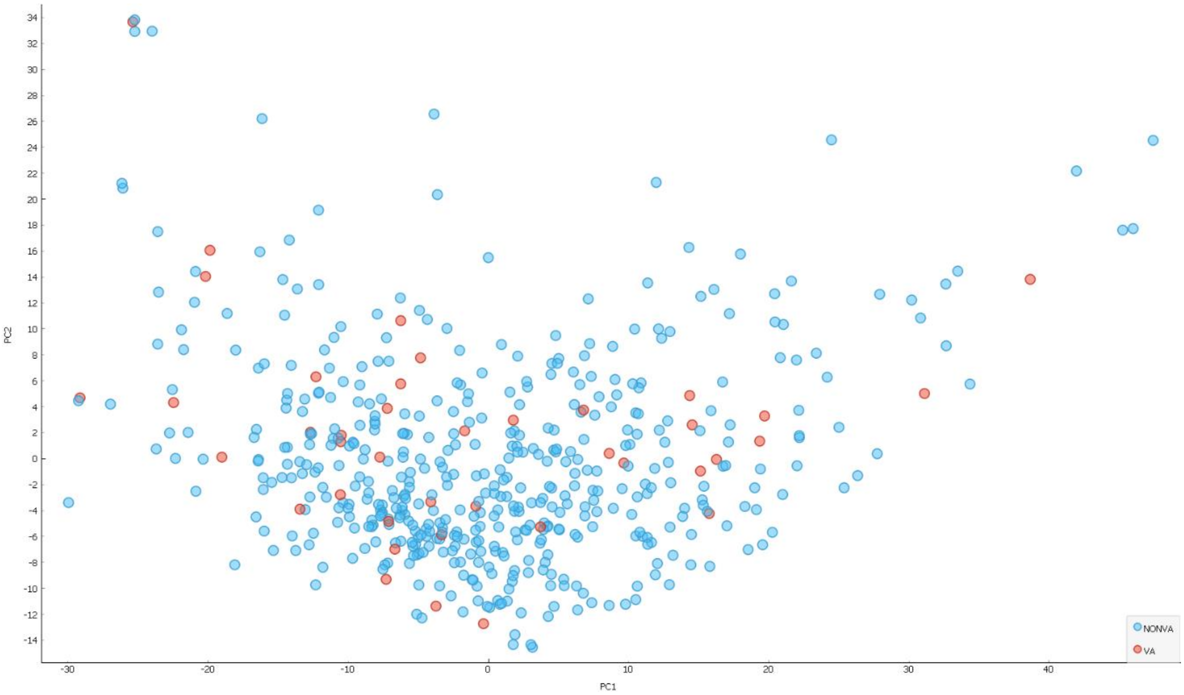

Figure S2: Principal component analysis (PCA) of dataset. Graph depicts arrangement of objects in multivariable space along the two axes with the largest variance. Principal component 1 (PC1) and principal component 2 (PC2) cover 37% of variance in the dataset. Red dots depict 40 objects in external validation set (VA) used in the second approach, blue dots depict 484 remaining objects (NONVA).

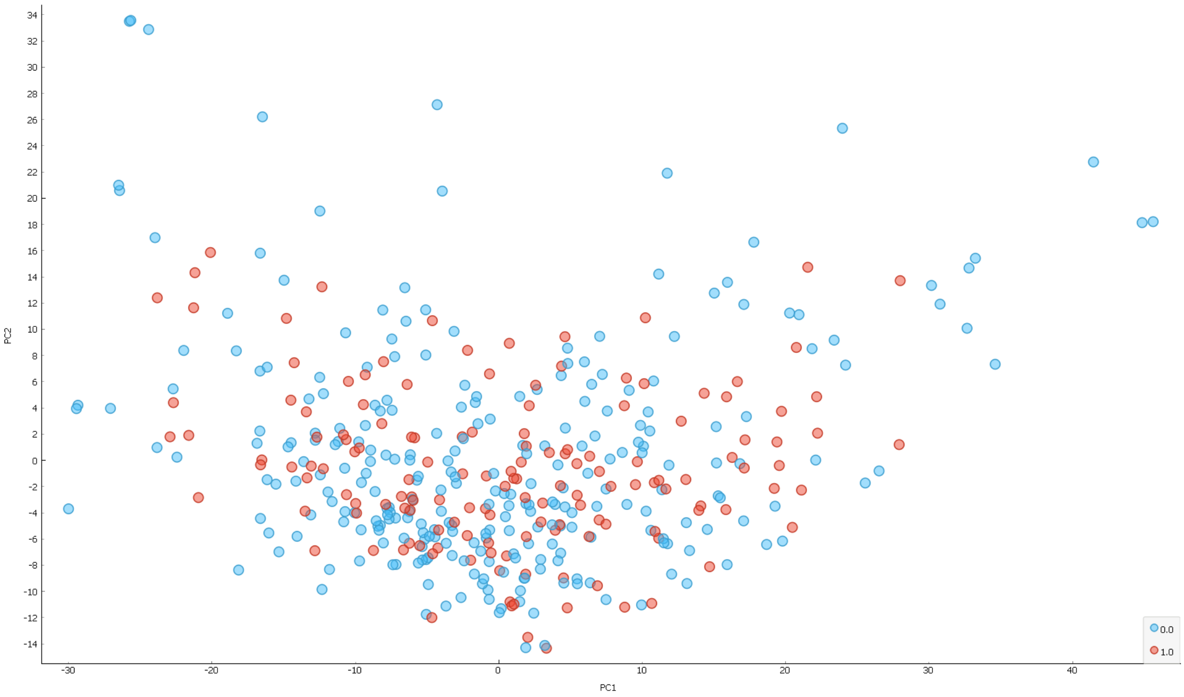

Figure S3: PCA on dataset of 422 objects after exclusion of validation set of 102 objects from initial dataset. Red dots (1.0) depict hepatotoxic compounds, blue dots (0.0) depict non-hepatotoxic compounds. First two principal components (PC1 and PC2) cover only 37% of variance in the depicted data.

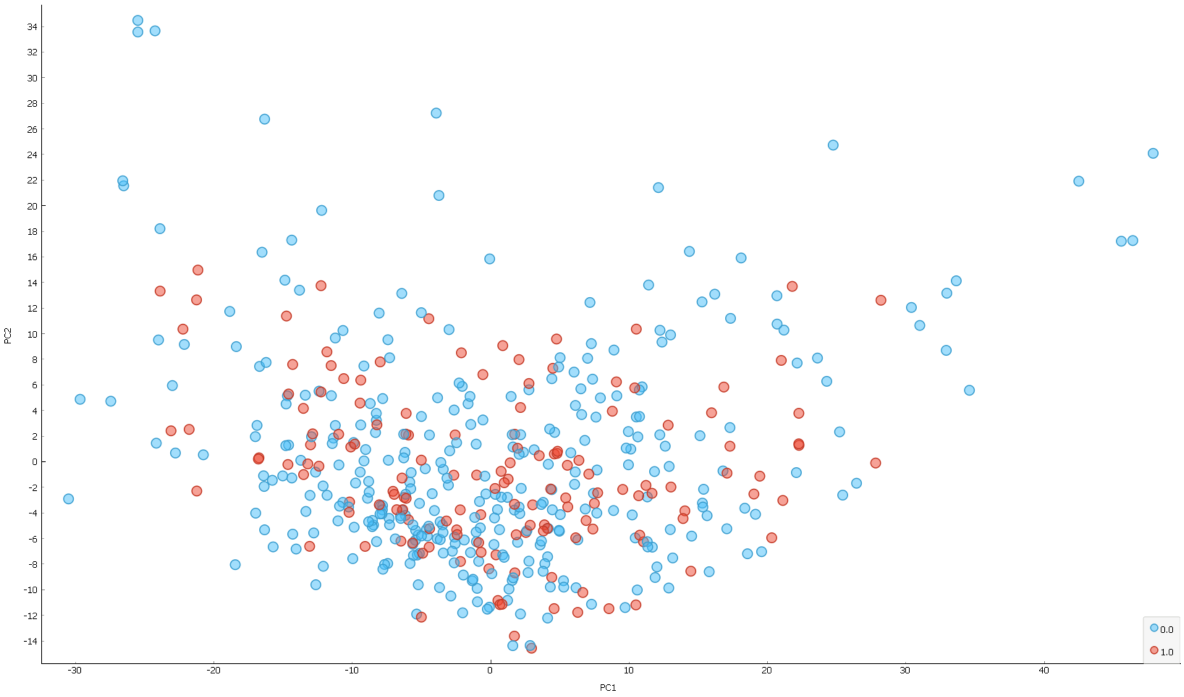

Figure S4: PCA on dataset of 484 objects after exclusion of validation set of 40 objects from initial dataset. Red dots (1.0) depict hepatotoxic compounds, blue dots (0.0) depict non-hepatotoxic compounds. First two principal components (PC1 and PC2) cover only 37% of variance in the depicted data.
